# Supplementary material for: Antigen specificity of clonally enriched CD8+ T cells in multiple sclerosis
Source: Nat Immunol. 2026 Feb 5;27(3):490–502. doi: 10.1038/s41590-025-02412-3 (PMC12956596; doi:10.1038/s41590-025-02412-3)
Supplement: Supplementary file 1 — Reporting Summary [file 41590_2025_2412_MOESM1_ESM.pdf]

nature portfolio

Corresponding author(s): Joseph J. Sabatino, Jr.

Last updated by author(s): Dec 8, 2025

Reporting Summary

Nature Portfolio wishes to improve the reproducibility of the work that we publish. This form provides structure for consistency and transparency in reporting. For further information on Nature Portfolio policies, see our Editorial Policies and the Editorial Policy Checklist.

Statistics

For all statistical analyses, confirm that the following items are present in the figure legend, table legend, main text, or Methods section.

n/a	Confirmed	
☐	☒	The exact sample size (*n*) for each experimental group/condition, given as a discrete number and unit of measurement
☒	☐	A statement on whether measurements were taken from distinct samples or whether the same sample was measured repeatedly
☐	☒	The statistical test(s) used AND whether they are one- or two-sided<br>*Only common tests should be described solely by name; describe more complex techniques in the Methods section.*
☒	☐	A description of all covariates tested
☐	☒	A description of any assumptions or corrections, such as tests of normality and adjustment for multiple comparisons
☐	☒	A full description of the statistical parameters including central tendency (e.g. means) or other basic estimates (e.g. regression coefficient) AND variation (e.g. standard deviation) or associated estimates of uncertainty (e.g. confidence intervals)
☐	☒	For null hypothesis testing, the test statistic (e.g. *F*, *t*, *r*) with confidence intervals, effect sizes, degrees of freedom and *P* value noted<br>*Give P values as exact values whenever suitable.*
☒	☐	For Bayesian analysis, information on the choice of priors and Markov chain Monte Carlo settings
☒	☐	For hierarchical and complex designs, identification of the appropriate level for tests and full reporting of outcomes
☒	☐	Estimates of effect sizes (e.g. Cohen's *d*, Pearson's *r*), indicating how they were calculated

Our web collection on statistics for biologists contains articles on many of the points above.

Software and code

Policy information about availability of computer code

Data collection	The software used for data collection is described in the relevant portions of the Materials and Methods.
Data analysis	The software and their versions used for data analysis listed below and are described in the relevant portions of the Materials and Methods.<br>CellRanger v3.0.1 (scRNA-seq) and v3.1.0 (scTCR-seq)<br>Seurat v3.1.2-v4.3.0<br>Spliced Transcripts Alignment to a Reference (STAR) algorithm v2.5.1<br>SingleR v1.1.7<br>DoubletFinder v2.0.2<br>Immcantation v3.1.0<br>Change-O v0.4.6<br>GraphPad Prism v10.6.1<br><br>All custom code used for the analysis of data and generation of plots is available upon request.

For manuscripts utilizing custom algorithms or software that are central to the research but not yet described in published literature, software must be made available to editors and reviewers. We strongly encourage code deposition in a community repository (e.g. GitHub). See the Nature Portfolio guidelines for submitting code & software for further information.

Data

Policy information about availability of data

All manuscripts must include a data availability statement. This statement should provide the following information, where applicable:

- Accession codes, unique identifiers, or web links for publicly available datasets
- A description of any restrictions on data availability
- For clinical datasets or third party data, please ensure that the statement adheres to our policy

All scRNA-seq data is available on BioProject PRJNA549712 (GEO accession no. GSE133028). All scTCR-seq data will be made publicly available without restrictions on BioProject PRJNA1232831 effective January 1, 2026 (GEO accession no. GSE291328).

Research involving human participants, their data, or biological material

Policy information about studies with human participants or human data. See also policy information about sex, gender (identity/presentation), and sexual orientation and race, ethnicity and racism.

Reporting on sex and gender	Sex data refers to biologic attributes of research participants.
Reporting on race, ethnicity, or other socially relevant groupings	N/A
Population characteristics	Age, sex, diagnosis, MRI and CSF information, and treatment status were provided in Table 1 and Supplemental Table 1 of the Manuscript.
Recruitment	MS/CIS and control participants were enrolled through the University of California San Francisco (UCSF) ORIGINS or Expression, Proteomics, Imaging, Clinical (EPIC) studies (https://epicstudy.ucsf.edu/). This study is designed to enroll patients early after experiencing an acute CNS demyelinating event. Healthy controls and OND patients were enrolled in the biobanking study "Immunological Studies of Neurologic Subjects". Informed consent was obtained from all participants in this study. No compensation was provided to study participants. We are not aware of any self-selection bias which would alter the results of this study as all patients and controls meeting eligibility criteria were able to enroll.
Ethics oversight	The studies in this manuscript have been approved by the UCSF IRB research ethics committee (protocol numbers 10-02389 and 14-15278).

Note that full information on the approval of the study protocol must also be provided in the manuscript.

Field-specific reporting

Please select the one below that is the best fit for your research. If you are not sure, read the appropriate sections before making your selection.

☒ Life sciences ☐ Behavioural & social sciences ☐ Ecological, evolutionary & environmental sciences

For a reference copy of the document with all sections, see nature.com/documents/nr-reporting-summary-flat.pdf

Life sciences study design

All studies must disclose on these points even when the disclosure is negative.

Sample size	Sample size was determined by sample availability. No sample size calculations were performed. Given the disproportionate number of participants in different disease categories, we grouped MS and CIS patients together (n = 13) and against OND and HC together as a comparison non-MS group (n = 5).
Data exclusions	No data were excluded. Specific criteria were employed for quality control and analysis of scRNA-seq and scTCR-seq data as described in Materials and Methods.
Replication	All flow cytometry experiments were performed in a minimum of two independent experiments. ddPCR experiment results were performed in duplicate.
Randomization	Samples were allocated based on clinical disease category.
Blinding	Researchers were not blinded during sample acquisition and data analysis as it was important for the investigators to know the disease status of the subjects in order to complete the data analysis.

Reporting for specific materials, systems and methods

We require information from authors about some types of materials, experimental systems and methods used in many studies. Here, indicate whether each material, system or method listed is relevant to your study. If you are not sure if a list item applies to your research, read the appropriate section before selecting a response.

Materials & experimental systems

n/a	Involved in the study
☐	☒ Antibodies
☐	☒ Eukaryotic cell lines
☒	☐ Palaeontology and archaeology
☒	☐ Animals and other organisms
☒	☐ Clinical data
☒	☐ Dual use research of concern
☒	☐ Plants

Methods

n/a	Involved in the study
☒	☐ ChIP-seq
☐	☒ Flow cytometry
☒	☐ MRI-based neuroimaging

Antibodies

Antibodies used

Anti-human CD8 PECy7 (eBioscience; SK1), anti-human TCR BV421 (BioLegend; IP26), anti-human CD4 PerCP-Cy5.5 (BioLegend; RPA-T4), anti-CD14 PerCP-Cy5.5 (BioLegend; HCD14), anti-human CD16 PerCP-Cy5.5 (BioLegend; B73.1), anti-human CD19 PerCP-Cy5.5 (BioLegend; HIB19), anti-human IFNg Alexa 647 (BioLegend; 4S.B3), anti-human TNFα Alexa 488 (BioLegend; Mab11), and anti-human CD69 PE (BioLegend; FN50). All antibodies were used at 1:100 dilutions.

Validation

All antibodies were purchased from the indicated commercial vendors with validation data and applicable citations available on product listings.

Eukaryotic cell lines

Policy information about cell lines and Sex and Gender in Research

Cell line source(s)

Jurkat E6-1 T cells (ATCC TIB-152)
293T cell line (ATCC CRL-3216)
K562 cell line (ATCC CCL-243)
Lymphoblastoid cell lines (LCLs) - EBV-transformed primary B cells from MS patients or healthy controls.

Authentication

None of the cell lines were authenticated.

Mycoplasma contamination

The cell lines were not tested for mycoplasma contamination.

Commonly misidentified lines
(See ICLAC register)

K562 cells

Plants

Seed stocks

N/A

Novel plant genotypes

N/A

Authentication

N/A

Flow Cytometry

Plots

Confirm that:

☒ The axis labels state the marker and fluorochrome used (e.g. CD4-FITC).

☒ The axis scales are clearly visible. Include numbers along axes only for bottom left plot of group (a 'group' is an analysis of identical markers).

☒ All plots are contour plots with outliers or pseudocolor plots.

☒ A numerical value for number of cells or percentage (with statistics) is provided.

Methodology

Sample preparation	For pMHC I tetramer analysis: Primary human CD8+ T cells expressing TCRs of interest were generated by CRISPR knockin as described in Materials and Methods. CD8+ T cells were treated wisdfsdfdth 100 nM dasatinib (StemCell) for 30 min at 37 °C followed by staining with the appropriate tetramers (2-3 µg/mL) for 30 min at room temperature. Cells were washed in FACS buffer and stained with the indicated cell surface antibodies for 30 minutes at 4°C. Cells were then washed and resuspended in FACS buffer and analyzed by flow cytometry.<br><br>For intracellular cytokine staining: APCs for were pulsed with 10 µg/ml peptide or vehicle control overnight in serum-free media. CD8+ T cells (2 x 105) were stimulated with peptide-loaded APCs (1 x 105 per condition) for 6 hours in the presence of 1:500 GolgiStop (BD), 1:500 GolgiPlug (BD), and 1:200 CD28/CD49d (FastImmune; BD). Cells were washed with FACS buffer and stained with the indicated cell surface antibodies for 30 minutes at 4°C. Cells were washed, fixed, and stained with anti-human IFNg Alexa 647 and anti-human TNFα Alexa 488 in permeabilization buffer (BD). Cells were then washed and collected on an LSRFortessa.<br><br>For Jurkat assays: TCR-expressing Jurkats were stimulated for 24 hours with HLA allele transduced APCs loaded with 10 µg/ml peptide or vehicle control. Antigen-reactive CD8+ cells were identified by co-expression of NFAT-mCherry and anti-human CD69 PE.
Instrument	BD LSRFortessa
Software	BD FACSDiva v9.0 was used for sample collection.<br>Flowjo v10.10.0 was used for analysis.
Cell population abundance	No sorting was performed in this study.
Gating strategy	Lymphocytes were identified by FSC-A/SSC-A followed by singlet gating using FSC-H/FSC-W. CD8+ T cells were selected against CD14/CD16/CD19 dump channel negative cells followed by live cell selection. After gating on TCR+ expressing CD8+ T cells, antigen-specific CD8+ T cells were identified by pMHC tetramer dual positivity (PE/APC) or intracellular cytokine production (IFNg vs TNFa). After gating on live CD8+ T cells in TCR-expressing Jurkats, antigen reactivity was determined by dual positivity of NFAT-mCherrry and CD69 PE.

☒ Tick this box to confirm that a figure exemplifying the gating strategy is provided in the Supplementary Information.
