# Supplementary figures and images for: Antigen specificity of clonally enriched CD8+ T cells in multiple sclerosis
Source: Nat Immunol. 2026 Feb 5;27(3):490–502. doi: 10.1038/s41590-025-02412-3 (PMC12956596; doi:10.1038/s41590-025-02412-3)

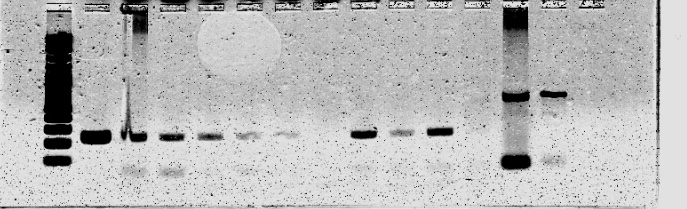

Supplement: Supplementary file 25 — Unprocessed gel of PCR. [file 41590_2025_2412_MOESM25_ESM.jpg]
